# Imaging features associated with idiopathic normal pressure hydrocephalus have high specificity even when comparing with vascular dementia and atypical parkinsonism

**DOI:** 10.1186/s12987-021-00270-3

**Published:** 2021-07-29

**Authors:** David Fällmar, Oliver Andersson, Lena Kilander, Malin Löwenmark, Dag Nyholm, Johan Virhammar

**Affiliations:** 1 Department of Surgical Sciences, Radiology, Uppsala University, Bonadsv 27, 75757 Uppsala, Sweden; 2 Department of Neuroscience, Neurology, Uppsala University, Uppsala, Sweden; 3 Department of Public Health and Caring Sciences, Geriatrics, Uppsala University, Uppsala, Sweden

**Keywords:** Normal pressure hydrocephalus, Differential diagnostics, Imaging features, Vascular dementia, Atypical parkinsonism

## Abstract

**Background:**

Vascular dementia (VaD) and atypical parkinsonism often present with symptoms that can resemble idiopathic normal pressure hydrocephalus (iNPH) and enlarged cerebral ventricles, and can be challenging differential diagnoses. The aim was to investigate frequencies of imaging features usually associated with iNPH and their radiological diagnostic accuracy in a sample containing the relevant differential diagnoses VaD, progressive supranuclear palsy (PSP), multiple system atrophy parkinsonian type (MSA-P), and healthy controls.

**Methods:**

Nine morphological imaging features usually associated with iNPH were retrospectively investigated in MR images of 55 patients with shunt-responsive iNPH, 32 patients with VaD, 30 patients with PSP, 27 patients with MSA-P, and 39 age-matched healthy controls. Logistic regression and receiver operating characteristic curves were used to assess diagnostic accuracy, sensitivity, and specificity for each imaging finding.

**Results:**

In a logistic regression model using iNPH diagnosis as a dependent variable, the following imaging features contributed significantly to the model: callosal angle (OR = 0.95 (0.92–0.99), p = 0.012), Evans’ index * 100 (OR = 1.51 (1.23–1.86), p < 0.001), enlarged Sylvian fissures (OR = 6.01 (1.42–25.40), p = 0.015), and focally enlarged sulci (OR = 10.18 (1.89–55.02), p = 0.007). Imaging features with 95% specificity for iNPH were: callosal angle ≤ 71°, temporal horns ≥ 7 mm, Evans’ index ≥ 0.37, iNPH Radscale ≥ 9, and presence of DESH, bilateral ventricular roof bulgings or focally enlarged sulci. A simplified version of the iNPH Radscale with only four features resulted in equally high diagnostic accuracy as the original iNPH Radscale.

**Conclusions:**

There is a notable overlap between some of the commonly used imaging markers regarding iNPH, VaD and atypical parkinsonism, such as PSP. However, this study shows that the specificity of imaging markers usually associated with iNPH was high even when comparing with these challenging differential diagnoses. The callosal angle was the single imaging feature with highest diagnostic accuracy to discriminate iNPH from its mimics. A simplified rating scale using only a few selected features could be used with retained specificity.

**Supplementary Information:**

The online version contains supplementary material available at 10.1186/s12987-021-00270-3.

## Background

Idiopathic normal pressure hydrocephalus (iNPH) is characterized by cognitive decline, gait disturbance, and urinary incontinence [1, 2]. The condition is increasingly recognized and prevalence is higher than previously believed [3, 4]. Symptoms can be reversed through implantation of a shunt system, with improvement in 50–80% of patients [5, 6]. Correct understanding of the imaging features of iNPH can potentially shorten doctor’s delay and time to shunt surgery.

Characteristic imaging findings in iNPH have been described as enlarged ventricles, effaced sulci at the high cerebral convexity, a sharp corpus callosal angle, and enlarged Sylvian fissures [7–10]. The recently published iNPH Radscale summarizes seven imaging features into a semiquantitative scale that has been validated in several studies [11–13]. The diagnostic accuracy of imaging features in iNPH has mainly been investigated in comparison to healthy controls (HC) or patients with Alzheimer’s disease [14, 15]. Since the typical morphological changes in Alzheimer’s disease differ from the typical findings in iNPH, the diagnostic accuracy of imaging features tested in that context would be high. Also, the neurological symptoms in Alzheimer’s disease often present differently than in iNPH. Frontotemporal degeneration and lewy-body dementia can present with both motor symptoms and cognitive symptoms but usually have other distinguishing features. In the clinical setting, vascular dementia (VaD) with or without motor symptoms, and atypical parkinsonian disorders, such as progressive supranuclear palsy (PSP) and multiple system atrophy of the parkinsonian type (MSA-P), can be even more plausible and relevant differential diagnoses than primary neurodegenerative dementia. Any addition to our ability to distinguish iNPH from its mimics would improve the clinical management and allow for a more correct selection of patients for beneficial surgical treatment.

The most well-known imaging feature of hydrocephalus is enlarged ventricles with Evans’ index > 0.3, but this is unspecific due to being common among the elderly [16]. Disproportionately enlarged subarachnoid-space hydrocephalus (DESH) is another morphological feature, used to refer to the combination of enlarged Sylvian fissures and narrow high-convexity sulci in the presence of hydrocephalus [10]. DESH has been described as a hallmark sign of iNPH [17], but is not present in all patients [9], and its frequency in other conditions is insufficiently described. Volumetric studies have shown that the pattern of compression and dilatation of CSF spaces can differentiate vascular and Alzheimer dementias from iNPH [18], but this has not yet been widely implemented in clinical routine. A need remains to investigate the frequencies of typical iNPH imaging features in other relevant differential diagnoses that can present with symptoms similar to those seen in iNPH.

The aims of this study were to investigate the frequency of nine iNPH-associated imaging features in patients with VaD, PSP, or MSA-P, and HC, and to find discriminating cut-off values between iNPH and these clinically relevant differential diagnoses.

## Methods

### Patients and controls

Inclusion criteria for iNPH patients were shunt surgery at a single university hospital during 2011–2015, improvement 12 months after surgery, defined as ≥ 5 points increase on the iNPH scale [19] (cognitive domain excluded) [20], and any preoperative MRI of the brain including a 3D sequence. An exclusion criterion was inadequate image quality for the relevant measurements. Patients charts were retrospectively reviewed by a neurologist (JV) to ascertain the diagnostic criteria of iNPH according to international guidelines [21], excluding other causes of hydrocephalus and neurodegenerative disorders. There was an overlap with previous studies that also included patients with iNPH from the same center during the time period [11, 20].

Patients with differential diagnoses (VaD, PSP, MSA-P) were identified retrospectively using diagnosis registers. Inclusion criteria were: clinical neurological evaluation during 2010–2018, including an MRI. Upon inclusion, diagnoses were confirmed through additional review of the charts by senior consultant specialists (LK, ML, DN) with expertise in each condition, using relevant diagnostic criteria [22–24]. Exclusion criteria were: inadequate image quality, two or more coexisting neurodegenerative disorders investigated in this study, or treatment with ventriculoperitoneal shunt for suspected iNPH.

Forty-five patients with VaD, 33 patients with PSP, and 31 patients with MSA-P were identified based on inclusion criteria, with 13, 2, and 3 patients in the respective groups excluded after review of patient charts by an expert. One PSP patient and one MSA-P patient were excluded because of shunt surgery for suspected iNPH. Age-matched HC were included from previous prospective studies. Exclusion criteria for HC were any known neurologic disease, stroke, diabetes mellitus, previous myocardial infarction with acute treatment or electrocardiogram changes, dependence on walking aids, or any terminal disease. Antihypertensive medication, aspirin, or common pain medications were allowed. For further details regarding inclusion criteria, please see the Supplement (Additional file 1). The study was approved by the National Ethical Review Authority (Dnr 2015/174/2).

### Imaging features

Nine imaging features were assessed based on written instructions (including definitions of planes for measurements and all semi-quantitative steps) made available to all investigators and discussed for disambiguation prior to assessment. The nine features were: Evans’ index, compression of (tight) high-convexity sulci, enlargement of Sylvian fissures, presence of focally enlarged sulci, width of temporal horns, callosal angle, periventricular hyperintensities (PVH), deep white matter hyperintensities (DWMH), and ventricular roof bulgings. The first seven features constitute the iNPH Radscale and the current assessments were made in line with the definitions provided in the original publication of that scale [11]. Deep white matter hyperintensities were visually assessed separately from periventricular hyperintensities and graded in accordance with Fazekas’ scale [25]. Confluent hyperintensities involving both deep and periventricular regions were primarily considered PVH. Ventricular bulgings were assessed as single/multiple, uni-/bilaterally, with at least 3 mm depth, in accordance with a previous publication [26]. Presence of DESH was defined as a combination of Sylvian fissure enlargement and high-convexity sulcal compression in patients with Evans’ index > 0.3.

Assessments were performed retrospectively on clinically acquired images, without strict conformity of scanner and imaging protocol. Morphological measurements and assessments were primarily made on 3DT1 images, and correlated with 3D FLAIR images in certain cases with motion artefacts. All morphological assessments were made in MPR mode, allowing for individual reconstruction along, and perpendicular to, the central bi-commissural plane used for all iNPH Radscale elements, including the callosal angle. White matter hyperintensities were assessed on 2D or 3D Flair images. Investigators were blinded to clinical data during assessments. Continuous features were measured by one investigator (OA, medical intern in training) and ordinal or dichotomous features were assessed by two investigators (OA + JV, > 10 years’ experience in iNPH-related radiology). A third investigator (DF, specialist in neuroradiology with > 10 years’ experience in iNPH-related radiology) also made an assessment when their assessments differed and the result with two votes was used in the statistical analysis. For the purpose of calculating interrater reliability, all imaging features were assessed by two investigators (OA and JV) in 30 randomly selected patients from different diagnostic groups (19 patients for Evans’ index and temporal horns).

### Statistics

Evans’ index was multiplied by 100 to facilitate statistical calculations [26]. Between-group differences were tested with Mann–Whitney U tests for continuous variables and χ^2^ tests and Fisher’s exact tests for ordinal and nominal data, respectively. Univariate logistic regression was used to calculate odds ratios (ORs) for discriminating between iNPH and each control group. A multivariable forward likelihood ratio model was used to test which imaging variables contributed significantly to the model. Receiver operating characteristic curves were used to assess sensitivity and specificity in discriminating iNPH from each condition. Interrater reliability was calculated with intraclass correlation coefficients for continuous data, weighted kappa for ordinal data, and Cohen’s kappa for nominal data. P-values < 0.05 were considered significant. Statistical analysis was performed using SPSS version 27 (IBM Corp, Armonk, NY).

## Results

Fifty-five patients with iNPH, 32 with VaD, 30 with PSP (19 probable, 11 possible), 27 with MSA-P (19 probable, 8 possible), and 39 HC were included in the statistical analysis. Demographics are presented in Table 1. Twenty-one (78%) of the patients with MSA-P, 16 (53%) of the patients with PSP and 2 (6%) of the patients with VaD were investigated with DatScan or PET during work-up.

**Table 1:** Demographics and frequencies of dichotomous imaging markers

	iNPH, n = 55	VaD, n = 32	PSP, n = 30	MSA-P, n = 27	HC, n = 39	p-value
Age (years), median (range)	71 (56–86)	74 (60–84)	73 (61–84)	66 (45–80)	73 (58–84)	0.003^a^
Sex, male	29 (53)	21 (66)	15 (50)	12 (44)	15 (39)	ns^b^
Focally enl. sulci	27 (49)	2 (6)	0 (0)	3 (11)	0 (0)	< 0.001^b^
Enl. Sylvian fissures	41 (75)	6 (19)	3 (10)	4 (15)	2 (5)	< 0.001^b^
DESH	35 (64)	4 (13)	2 (7)	0 (0)	0 (0)	< 0.001^b^
EI > 0.3	54 (98)	16 (50)	18 (60)	10 (37)	9 (23)	< 0.001^b^

All data except age are n (%)

iNPH, idiopathic normal pressure hydrocephalus; VaD, vascular dementia; PSP, progressive supranuclear palsy; MSA-P, multiple system atrophy parkinsonian type; HC, healthy controls; enl., enlarged; DESH, disproportionately enlarged subarachnoid-space hydrocephalus; EI, Evans’ index; ns, not significant

^a^Kruskal Wallis. Mann–Whitney was used as a post hoc test between each group and revealed that there was a significant difference in age between MSA-P and all other groups (iNPH vs MSA-P, p = 0.005)

^b^χ^2^ test. All imaging markers were more frequent in iNPH than in the other groups

**Fig. 1:**
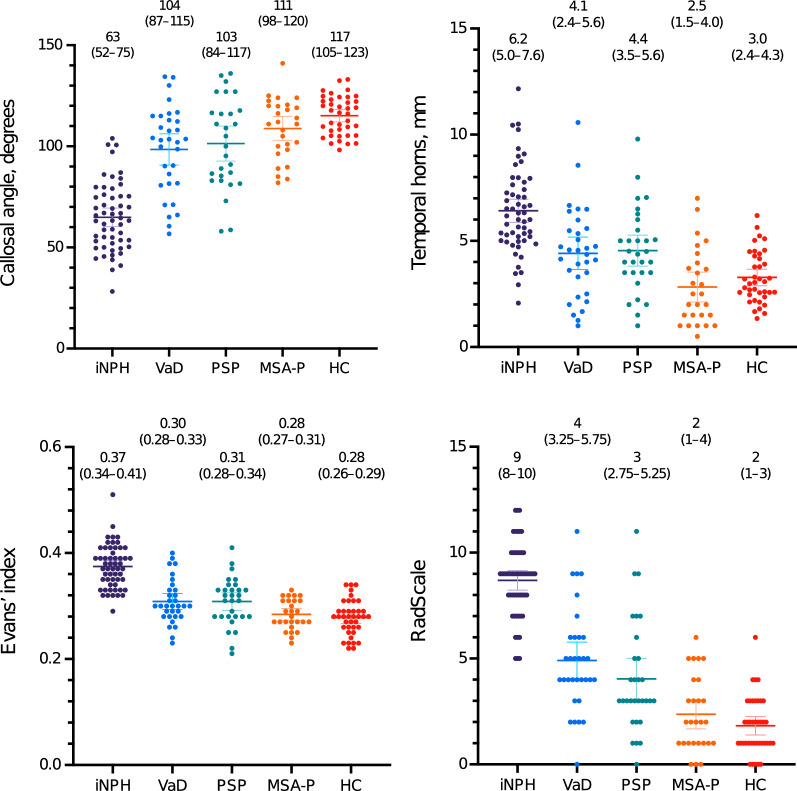
Median and error bars representing 25th and 75th percentile. Each point is one patient/control. Data at the top are medians (interquartile ranges). All four imaging markers are significantly different between iNPH and each control group (p < 0.001). iNPH = idiopathic normal pressure hydrocephalus; PSP = progressive supranuclear palsy; VaD = vascular dementia; MSA-P = multiple system atrophy parkinsonian type; HC = healthy controls

**Fig. 2:**
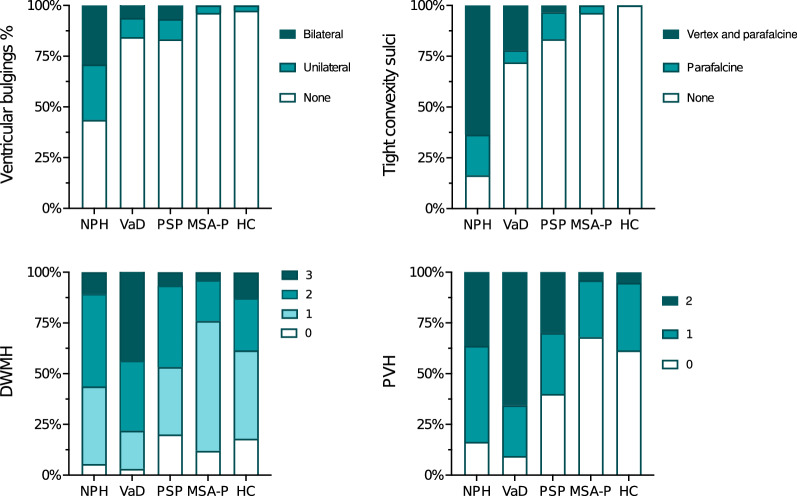
Frequency of ventricular roof bulgings, deep white matter hyperintensities (DWMH), periventricular hyperintensities (PVH), and tight sulci in patients with idiopathic normal pressure hydrocephalus (NPH), vascular dementia (VaD), progressive supranuclear palsy (PSP), multiple system atrophy parkinsonian type (MSA-P), and healthy controls (HC)

All continuous radiological markers and the semiquantitative iNPH Radscale sums were significantly different in iNPH compared with HC and each differential diagnosis, as shown in Fig. 1, with p-values consistently < 0.001. Tight sulci and ventricular bulgings were more frequently present in iNPH than in the other groups (p < 0.001), Fig. 2. PVH were more often present in iNPH than in all groups except VaD (p < 0.001 to p = 0.049). PVH was more pronounced in VaD than iNPH (p = 0.014). High grades of DWMH were more common in patients with VaD than in iNPH (p = 0.002) and there was no difference in frequency of DWMH between iNPH and the other groups (Fig. 2).

Focally enlarged sulci were present in 49% of patients with iNPH, enlarged Sylvian fissures in 75%, and DESH in 64%. These findings were rare in HC (≤ 5%) and uncommon in the differential diagnoses (0–19%), Table 1.

Odds ratios for univariate logistic regression for all variables are presented in Table 2. Callosal angle was the single marker with the highest area under the receiver operating characteristic curve (AUC) (0.94) for discriminating iNPH from a combined group of both HC and the differential diagnoses, while AUC was 0.95 for iNPH Radscale score. In a multivariable logistic regression model using forward likelihood ratio, only the markers callosal angle, Evans’ index, focally enlarged sulci and enlarged Sylvian fissures contributed significantly to the model (Table 2). Using these four markers, a simplified version of the iNPH Radscale was calculated for each patient, resulting in an AUC of 0.96, slightly higher than the original iNPH Radscale with seven features.

**Table 2:** Odds ratios for discrimination of patients with iNPH from a group of patients with vascular dementia, progressive supranuclear palsy, multiple system atrophy, and healthy controls

Univariate				Multivariable	
	OR (95% CI)	p-value	AUC	OR	p-value
Callosal angle	0.90 (0.88–0.93)	< 0.001	0.94	0.95 (0.92–0.99)	0.012
Temporal horns	1.96 (1.57–2.45)	< 0.001	0.84		
Evans’ index (*100)	1.61 (1.40–1.85)	< 0.001	0.93	1.51 (1.23–1.86)	< 0.001
Tight sulci	7.78 (4.62–13.08)	< 0.001	0.87		
Vent. bulgings	5.36 (3.0–9.59)	< 0.001	0.74		
Focally enl. sulci	23.72 (8.40–67.03)	< 0.001	0.73	10.18 (1.89–55.02)	0.007
Sylvian fissure	22.06 (9.80–49.66)	< 0.001	0.81	6.01 (1.42–25.40)	0.015
DESH	35.58 (13.27–95.45)	< 0.001	0.80		
PVH	1.83 (1.22–2.76)	0.004	0.63		
DWMH	1.14 (0.80–1.64)	0.47	0.54		
Radscale	2.48 (1.92–3.22)	< 0.001	0.95		
Simplified Radscale	7.16 (3.97–12.92)	< 0.001	0.96		

iNPH, idiopathic normal pressure hydrocephalus; DESH, disproportionately enlarged subarachnoid-space hydrocephalus; OR, odds ratio; CI, confidence interval; AUC, area under the receiver operating characteristic curve; Vent., ventricular; enl., enlarged; PVH, periventricular hyperintensities; DWMH, deep white matter hyperintensities

Each marker was tested with univariate logistic regression and a multivariable logistic regression model using forward likelihood ratio including all imaging markers except DESH and iNPH Radscale (which are combinations of several markers). The odds ratio is < 1 for Callosal angle, meaning that a higher Callosal angle is associated with lower probability of shunt responsive iNPH. The odds ratio for Evans’ index (*100) represents an increase of the variable by 0.01

**Fig. 3:**
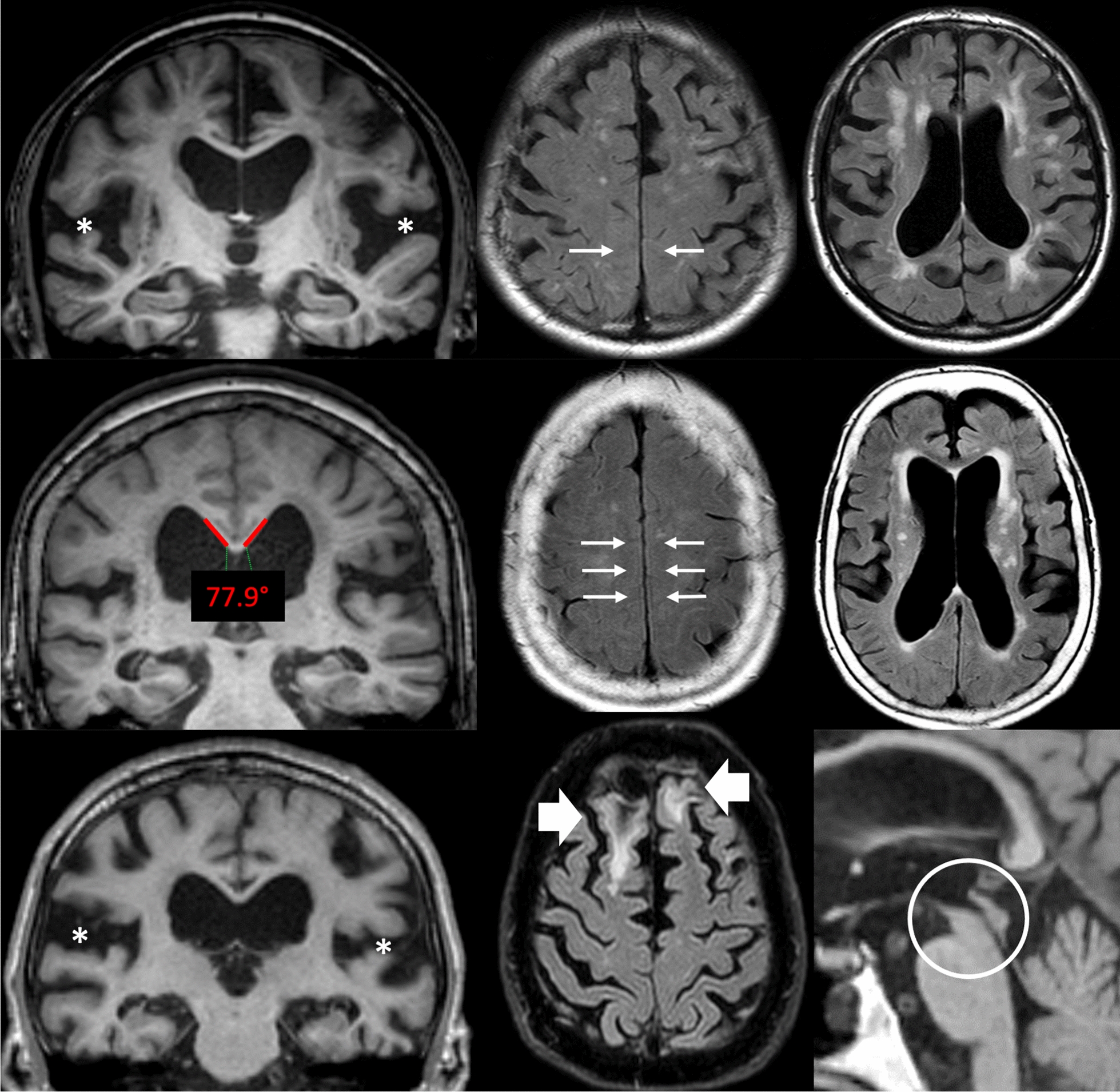
Images from three patients, highlighting some of the difficulties of differential diagnostics. The top row shows a patient with vascular dementia, but also disproportionately widened Sylvian fissures (asterisks) and compression of high-convexity sulci (arrows). The white matter changes are predominantly in deep white matter. The middle row shows another patient with a clinical diagnosis of vascular dementia. This patient has a callosal angle of 77.9°, widespread compression of high-convexity sulci (arrows) and white matter changes that are mostly periventricular. Vascular dementia and idiopathic normal pressure hydrocephalus (iNPH) can hardly be distinguished in this situation. The bottom row shows a patient with progressive supranuclear palsy with general atrophy and wide cerebrospinal fluid spaces, including the Sylvian fissures (asterisks). Parenchymal defects after previous trauma (wide arrows) clouded the neurological assessment. Although decreased mesencephalic area is a common finding in iNPH, severe atrophy (circle) would imply progressive supranuclear palsy. All three patients had 9 points on the iNPH Radscale

Sensitivity and specificity for different cut-offs of the imaging markers are presented in Table 3. The specificity was higher for all markers when HC were added to the combined group of differential diagnoses. Specificity for discriminating iNPH from differential diagnoses (with HC excluded) was 88% for the presence of DESH, and 94% for callosal angle < 63°. VaD was the differential diagnosis with most overlap with iNPH, Table 3. Differential diagnostic aspects in three cases with iNPH-associated imaging features are illustrated in Fig. 3.

**Table 3:** Sensitivity and specificity for presence of imaging features and some predefined cut-offs to discriminate shunt-responsive iNPH from each control group (the first four columns), and from a combination of all differential diagnoses without or with healthy controls (the last two columns)

	VaD	MSA-P	PSP	HC	VaD + MSA-P + PSP	VaD + MSA-P + PSP + HC
Callosal angle < 90°	93/69	93/85	93/61	93/100	93/70	93/79
Callosal angle < 63°	46/94	47/100	47/93	47/100	47/95	47/97
Temporal horns ≥ 4 mm	91/41	91/78	91/47	91/78	91/54	91/59
Temporal horns ≥ 6 mm	55/81	55/93	55/77	55/97	55/83	55/88
Evans’ index > 0.3	98/50	98/67	98/40	98/77	98/51	98/59
Crowded sulci	84/72	84/96	84/83	84/100	84/83	84/88
Ventricular bulgings	56/84	56/96	56/83	56/97	56/88	56/91
Focally enlarged sulci	49/94	49/89	49/100	49/100	49/94	49/96
Sylvian fissure	75/81	75/85	75/90	75/95	75/85	75/88
DESH	64/88	64/100	64/93	64/100	64/93	64/95
PVH = 2	36/34	36/96	36/70	36/95	36/64	36/74
DWMH ≥ 2	56/22	56/76	56/53	56/62	56/48	56/52
Radscale ≥ 5	100/50	100/82	100/70	100/97	100/67	100/76
Radscale ≥ 8	78/84	78/100	78/90	78/100	78/92	78/95
Simplified Radscale ≥ 4	89/81	89/96	89/90	89/100	89/89	89/92

Data are sensitivity/specificity (%)

iNPH, idiopathic normal pressure hydrocephalus; VaD, vascular dementia; MSA-P, multiple system atrophy parkinsonian type; PSP, progressive supranuclear palsy; HC, healthy controls; DESH, disproportionately enlarged subarachnoid-space hydrocephalus; PVH, periventricular hyperintensities; DWMH, deep white matter hyperintensities

Cut-off values that resulted in a specificity > 95% for discriminating iNPH from all other control groups combined were: callosal angle ≤ 71° (sensitivity 69%); temporal horns ≥ 7 mm (sensitivity 35%); Evans’ index ≥ 0.37 (sensitivity 58%); bilateral ventricular bulgings (sensitivity 29%); focally enlarged sulci (sensitivity 49%); DESH (sensitivity 64%); iNPH Radscale score ≥ 9 (sensitivity 60%); simplified iNPH Radscale score ≥ 5 (sensitivity 53%).

### Reliability

The reliabilities of continuous variables, calculated with intraclass coefficient correlations, were: callosal angle 0.97, Evans’ index 0.99, temporal horns 0.91. Weighted kappa for ordinal data: ventricular bulging 0.46, DWMH 0.59, PVH 0.74. Kappa for nominal data: Sylvian fissures 0.90, focally enlarged sulci 0.76. All reliability measures were significant with p < 0.001, except ventricular bulgings with p = 0.003.

## Discussion

This study compared neuroimaging features associated with normal pressure hydrocephalus in patients with idiopathic normal pressure hydrocephalus, healthy controls, and the relevant differential diagnoses vascular dementia, progressive supranuclear palsy and multiple system atrophy parkinsonian type. Regarding discrimination of idiopathic normal pressure hydrocephalus from the other groups, odds ratios were significant for all tested imaging markers except periventricular hyperintensities and deep white matter hyperintensities. In a multivariable model, callosal angle, Evans’ index, enlarged Sylvian fissures, and focally enlarged sulci all contributed. The single feature with the highest diagnostic AUC was the callosal angle, which is well described in previous literature and has been shown to have good interrater agreement.

A noteworthy result was that Evans’ index was above 0.3 in a majority of patients with PSP, half of the patients with VaD and also present in 23% of the healthy controls. Enlarged ventricles is a diagnostic criterion for iNPH and Evans’ index is its most well-known descriptor [21]. In the current study, values above 0.37 were specific for iNPH (specificity > 95%), but in the range 0.30–0.37 a combination of other imaging features would be needed to confirm the radiological suspicion of iNPH.

Calculating odds ratios for Evans’ index can be handled in different manners. An increase of one (whole) unit in Evans´ index is clinically impossible and would produce astronomical numbers which would be difficult to interpret. In the current study, the index was multiplied by 100 (Table 2), which equals an increment of 0.01. On the other hand, 0.01 is a rather small unit of increase from a clinical perspective. Separate calculations were performed using an increment of 0.03 with similar results (data not shown).

Several of the evaluated features showed considerable overlap between iNPH and VaD and between iNPH and PSP. This could be explained by diagnostic complexity; it is also possible that some patients suffered from two conditions. There may even be shared pathophysiological mechanisms that are currently largely unexplored. The diagnostic challenge in this setting is demonstrated by the fact that one patient with PSP and one with MSA-P in this study were excluded because of concomitant iNPH diagnoses with ventriculoperitoneal shunt surgery. Moreover, in the absence of a previous clinical stroke, the VaD diagnosis can be challenging and relies heavily on radiological assessment [22]. However, it can be difficult to differentiate between white matter hyperintensities caused by small vessel disease (common in VaD) and PVH caused by transependymal passage of CSF (common in iNPH). In this study, there were PVH with appearance of excessive transependymal passage of CSF in four patients diagnosed with VaD. Comorbidity with iNPH cannot be excluded in these patients. If these four patients were excluded from statistical analysis the calculated specificities to discriminate between iNPH and VaD were even higher for most imaging features (data not shown). For clinical purposes, the overlap of imaging findings between diagnoses, especially between iNPH and VaD, should increase awareness that the cause of white matter hyperintensities on MRI can be difficult to interpret. VaD is indeed a challenging differential diagnosis to iNPH, both clinically and from a research perspective. This needs to be further examined in future studies—an additional MRI sequence that can differentiate chronic ischemic changes from subependymal oedema would provide essential information, for example.

Another relevant finding was the high diagnostic accuracy of the iNPH Radscale and even of a short version of the scale, using only four features. Semiquantitative rating scales (Scheltens, Fazekas, Koedam) have been successfully implemented in radiological work-up of suspected dementia and provide the referring physicians with useful data. In our clinical experience, the iNPH Radscale can be successfully implemented in the clinical work-up of suspected iNPH and provides a total score that represents the strength of the imaging-based likelihood for iNPH [12]. In the current study, a short version with only four items had similar diagnostic accuracy—an appealing concept that should be further validated in a separate context. Several of the imaging markers included in this manuscript each have high specificity but low sensitivity. This is interpreted as a confirmation of the notion that a combination of imaging features continues to be a valid biomarker strategy for iNPH.

As in previous studies, the interrater reliability was excellent for markers measured on a continuous scale, such as callosal angle and Evans’ index, but lower for markers graded on a subjective ordinal scale. This highlights that continuous imaging features may be preferable for implementation in clinical routine with only one rater available. Future decision support tools based on artificial intelligence and direct comparison to age-matched controls may strengthen and facilitate this further.

Limitations of this study include the retrospective design, which is susceptible to inclusion bias. Imaging was performed on different scanners with differing techniques, which introduces theoretical confounders due to slight differences in resolution and impact of field heterogeneities. Compared to the margin of errors during radiological assessments, along with non-perfect intra- and interrater agreements, the theoretical differences between scanners and sequences were considered to have negligible impact on the morphological results presented in this paper. Similarly, the sensitivity for white matter changes can differ slightly between 2D and 3D FLAIR, but the difference is small compared to the coarse semiquantification used in this study. The limited size of the study did not allow careful corrections for age and disease duration, that could have increased the clarity of the results. Another limitation of this study was the retrospective design and obvious uncertainty of the VaD-diagnosis in some cases. It is possible that some of the overlap between iNPH and VaD was caused by patients that had been misdiagnosed or actually had two conditions. Distinguishing between neurological disorders based on vascular pathology is generally insufficiently described in literature, but a common clinical issue. Assessment of shunt response in the iNPH patients was done without inclusion of cognitive tests which may have led to some inclusion bias since patients with only cognitive improvement after shunting were excluded. Inclusion of the full range of conditions encountered in clinical practice in the differential diagnosis of iNPH was beyond the scope of this study.

## Conclusions

Several imaging features associated with iNPH have previously been shown to have diagnostic accuracy and excellent ability to discriminate iNPH from healthy controls and Alzheimer’s disease. This study shows that several features, such as callosal angle, enlarged Sylvian fissures and focally enlarged sulci, maintain diagnostic accuracy also in comparison with relevant differential diagnoses, including vascular dementia. However, there is a notable overlap for some of the imaging markers between iNPH, vascular dementia and progressive supranuclear palsy. Callosal angle was the single imaging feature with highest accuracy to discriminate iNPH from its mimics. A simplified version of the iNPH Radscale using only four features could be used with retained specificity.

## Supplementary Information

**Additional file 1.** A supplement document with details regarding diagnostic inclusion criteria.

## Data Availability

The datasets analyzed during the current study are available from the corresponding author on reasonable request.

## Abbreviations

iNPH: Idiopathic normal pressure hydrocephalus
HC: Healthy controls
VaD: Vascular dementia
MSA-P: Multiple system atrophy parkinsonian type
PSP: Progressive supranuclear palsy
DESH: Disproportionately enlarged subarachnoid-space hydrocephalus
CSF: Cerebrospinal fluid
DWMH: Deep white matter hyperintensities
PVH: Periventricular hyperintensities
OR: Odds ratio
