# Supplementary material for: Imaging features associated with idiopathic normal pressure hydrocephalus have high specificity even when comparing with vascular dementia and atypical parkinsonism
Source: Fluids Barriers CNS. 2021 Jul 29;18:35. doi: 10.1186/s12987-021-00270-3 (PMC8323278; doi:10.1186/s12987-021-00270-3)
Supplement: Supplementary file 1 — Additional file 1. A supplement document with details regarding diagnostic inclusion criteria. [file 12987_2021_270_MOESM1_ESM.docx]

**Additional file 1 - diagnostic inclusion criteria**

Patients that had been diagnosed with more than one of the diagnoses investigated in this study were not included.

**iNPH**

Diagnosis was based on the international guidelines of iNPH [1] with the following modification: patients with no urinary symptoms and normal cognitive function (Mini-Mental State Examination 28-30) was included if the gait and balance dysfunction was considered typical of iNPH and there were other supportive features such as typical imaging, positive response to CSF tap test or increased resistance to outflow in the CSF dynamic studies.

Patients with a high-quality MR of the brain including a 3D sequence that were operated within the years 2011-2015 at Uppsala University Hospital with evaluations of symptoms before and 12 months after shunt surgery with ≥ 5 points improvement in a modified iNPH-scale were included. The cognitive domain of the iNPH-scale was not included.

**Vascular dementia**

Vascular dementia was defined according to modified ADDTC criteria [2], i.e. a significant cognitive decline interfering with everyday activities identified by history and cognitive testing, and with evidence of vascular origin according to history, i.e. a temporal relationship, and neuroimaging: either one single ischemic stroke with strategic localization or > 2 ischemic strokes outside the cerebellum and/or widespread white matter hyperintensities (Fazekas grade 3).

**MSA-P and PSP**

The diagnostic criteria for possible or prodromal MSA were used [3], based on clinical examination. For possible or probable PSP diagnosis, the clinical research criteria from 1996 were applied [4].

**Healthy controls**

Controls were included from two previous prospective studies [5,6] (Dnr: 2011/370/1 and Dnr: 2010/161). Exclusion criteria for HC were any known neurologic disease, stroke, diabetes mellitus, previous myocardial infarction with acute treatment or electrocardiogram changes, dependence on walking aids, or any terminal disease. Antihypertensive medication, aspirin, or common pain medications were allowed.

**References**

1. Relkin N, Marmarou A, Klinge P, Bergsneider M, Black PM (2005) Diagnosing idiopathic normal-pressure hydrocephalus. Neurosurgery 57 (3 Suppl):S4-16; discussion ii-v. doi:10.1227/01.neu.0000168185.29659.c5

2. Chui HC, Victoroff JI, Margolin D, Jagust W, Shankle R, Katzman R (1992) Criteria for the diagnosis of ischemic vascular dementia proposed by the State of California Alzheimer's Disease Diagnostic and Treatment Centers. Neurology 42 (3 Pt 1):473-480. doi:10.1212/wnl.42.3.473

3. Gilman S, Wenning GK, Low PA, Brooks DJ, Mathias CJ, Trojanowski JQ, Wood NW, Colosimo C, Dürr A, Fowler CJ, Kaufmann H, Klockgether T, Lees A, Poewe W, Quinn N, Revesz T, Robertson D, Sandroni P, Seppi K, Vidailhet M (2008) Second consensus statement on the diagnosis of multiple system atrophy. Neurology 71 (9):670-676. doi:10.1212/01.wnl.0000324625.00404.15

4. Litvan I, Agid Y, Calne D, Campbell G, Dubois B, Duvoisin RC, Goetz CG, Golbe LI, Grafman J, Growdon JH, Hallett M, Jankovic J, Quinn NP, Tolosa E, Zee DS (1996) Clinical research criteria for the diagnosis of progressive supranuclear palsy (Steele-Richardson-Olszewski syndrome): report of the NINDS-SPSP international workshop. Neurology 47 (1):1-9. doi:10.1212/wnl.47.1.1

5. Virhammar J, Laurell K, Ahlgren A, Larsson EM (2017) Arterial Spin-Labeling Perfusion MR Imaging Demonstrates Regional CBF Decrease in Idiopathic Normal Pressure Hydrocephalus. AJNR Am J Neuroradiol 38 (11):2081-2088. doi:10.3174/ajnr.A5347

6. Fallmar D, Haller S, Lilja J, Danfors T, Kilander L, Tolboom N, Egger K, Kellner E, Croon PM, Verfaillie SCJ, van Berckel BNM, Ossenkoppele R, Barkhof F, Larsson EM (2017) Arterial spin labeling-based Z-maps have high specificity and positive predictive value for neurodegenerative dementia compared to FDG-PET. Eur Radiol 27 (10):4237-4246. doi:10.1007/s00330-017-4784-1
